# Genomic bacterial load associated with bacterial genotypes and clinical characteristics in patients with scrub typhus in Hainan Island, Southern China

**DOI:** 10.1371/journal.pntd.0011243

**Published:** 2023-04-21

**Authors:** Gaoyu Wang, Ruijia Fu, Liyuan Zhang, Liying Xue, Abdullah Y. Al-Mahdi, Xiaofei Xie, Aiping Qin, Chuanning Tang, Jiang Du, Yi Huang, Yueping Wang, Jian Su, Shengkai Huang, Ruoyan Peng, Zhe Lu, Jing An, Changjia Sun, Hua Yang, Changhua He, Kwok-Yung Yuen, Jasper Fuk-Woo Chan, Yongguo Du, Meifang Xiao, Long Sun, Feifei Yin

**Affiliations:** 1 Hainan Medical University-The University of Hong Kong Joint Laboratory of Tropical Infectious Diseases, Key Laboratory of Tropical Translational Medicine of Ministry of Education, Hainan Medical University, Haikou, China; 2 Department of Clinical Laboratory, Center for Laboratory Medicine, Hainan Women and Children’s Medical Center, Haikou, China; 3 Academician Workstation of Hainan Province, Hainan Medical University; Haikou, China; 4 Department of Infectious Disease, the Second Affiliated Hospital of Hainan Medical University, Haikou, China; 5 Faculty of medicine, Lincoln University College, Petaling Jaya, Malaysia; 6 State Key Laboratory of Infectious Diseases Prevention and Control, National Institute for Communicable Disease Control and Prevention, Chinese Center for Disease Control and Prevention, Beijing, China; 7 Department of Infectious Disease, the First affiliated Hospital of Hainan Medical University, Haikou, China; 8 Hainan Center for Disease Control and Prevention, Haikou, China; 9 State Key Laboratory of Emerging Infectious Diseases, The University of Hong Kong, Pokfulam, Hong Kong, China; Postgraduate Institute of Medical Education and Research, INDIA

## Abstract

Scrub typhus, caused by mite-borne *Orientia tsutsugamushi* (*O*. *tsutsugamushi*), is a major febrile disease in the Asia-Pacific region. The DNA load of *O*. *tsutsugamushi* in the blood was previously found to be significantly higher in patients with fatal disease than those with non-fatal disease and correlated with the duration of illness, presence of eschar, and hepatic enzyme levels. In this prospective observation study, we analyzed the association of bacterial DNA load with clinical features, disease severity, and genotype using real-time PCR targeting the 56 kDa TSA gene of *O*. *tsutsugamushi* in the blood samples of 117 surviving patients with scrub typhus who had not received appropriate antibiotic treatment. The median *O*. *tsutsugamushi* DNA load was 3.11×10^3^ copies/mL (range, 44 to 3.3×10^6^ copies/mL). The severity of patients was categorized as mild, moderate, and severe based on the number of dysfunctional organs, and no significant difference in *O*. *tsutsugamushi* DNA load was found among these groups. Patients infected with the Karp group showed a significantly higher *O*. *tsutsugamushi* DNA load than those in the Gilliam (*P* < 0.05) and TA763 (*P* < 0.01) groups. Patients belonging to the Li ethnic group showed a significantly higher DNA load than those in the Han ethnic groups. The blood bacterial DNA load of patients showed no significant difference between groups divided by gender, age, with or without eschar, or the season of disease onset. The highest body temperature recorded during fever onset was positively correlated with *O*. *tsutsugamushi* DNA load (ρ  =  0.272, *P * =  0.022). Correlation analyses indicated that the serum total bilirubin level was positively correlated with *O*. *tsutsugamushi* DNA load. In conclusion, the findings in this study demonstrated the association of DNA load of *O*. *tsutsugamushi* with the severity and genotype in patients with scrub typhus in Hainan, China.

## Author summary

High genomic DNA load is a marker for the virulence of the pathogen and is associated with disease severity in bacterial infectious diseases. Recent studies have indicated that the non-surviving patients with scrub typhus had a significantly higher bacterial load than the surviving patients, and that duration of illness, presence of eschar, and hepatic enzyme levels were positively correlated with the *O*. *tsutsugamushi* DNA load in patient from northeast Thailand and South Korea. However, the severity and fatality of scrub typhus showed largely geographic variability. To further analyze the association of genomic DNA load with severity in surviving patients, the *O*. *tsutsugamushi* genomic load in blood samples of patients with scrub typhus in Hainan, China was evaluated. The results indicated that *O*. *tsutsugamushi* DNA load was associated with the genotypes, ethnic group of the patients, the highest body temperature recorded during fever onset and the total bilirubin (TBil) in the blood. However, no significant difference in *O*. *tsutsugamushi* DNA load was found between mild, moderate, and severe patient groups, nor between groups of patients with or without eschar. This research contributes to a better overall understanding of the pathogenic mechanism of *O*. *tsutsugamushi*.

## Introduction

*Orientia tsutsugamushi* (*O*. *tsutsugamushi*) is an obligate intracellular bacterium that causes the mite-borne acute febrile disease scrub typhus, which accounts for up to 20% of all febrile cases in the Asia-Pacific region (the Tsutsugamushi Triangle). The disease was also discovered in the Arabian Peninsula, Chile, and possibly Kenya, suggesting that it may be more widespread than previously thought [1–4]. The widespread re-emergence of scrub typhus in India, Micronesia, and the Maldives, and the rapid emergence of cases in northern China and South Korea have raised considerable concern in endemic countries [5,6]. The most common clinical features of scrub typhus are fever and headache beginning 7–14 days after inoculation with an infected larval mite. Other symptoms include rash, myalgia, cough, lymphadenopathy, nausea, vomiting, abdominal pain, and eschar at the site of mite bite [7–9]. Additionally, multiple organ failures including acute renal failure, acute respiratory distress, meningoencephalitis, myocarditis, septic shock, and disseminated intravascular coagulation have been reported in severe cases [10,11]. The fatality rate is 30–50% in untreated cases and 4% in cases treated with antibiotics [12,13]. *O*. *tsutsugamushi* is subdivided into 3 classes: high virulence (Karp, Kato, and KN-3 serotypes), intermediate virulence (Gilliam serotype), and low virulence (Kuroki, Kawasaki, and KN-2 serotypes) [14]. Although scrub typhus is a major public health issue, it remains a neglected disease worldwide [5].

The severity and fatality of scrub typhus show geographic variability [13]. The basis for this variability remains largely unknown and was proposed to be influenced by multiple factors, including differences in host genetic susceptibility and immune response, variability in the virulence of the pathogen, and vector species. Additionally, recent studies have investigated the relationship between *O*. *tsutsugamushi* genomic DNA load and clinical characteristics of scrub typhus. The results indicated that the patients with fatal disease had a significantly higher bacterial load than patients with non-fatal disease, and that duration of illness, presence of eschar, and hepatic enzyme levels were positively correlated with the *O*. *tsutsugamushi* DNA load [15,16] High genomic DNA load is a marker for the virulence of the pathogen and is associated with disease severity in bacterial infectious diseases. In severe sepsis, the genomic bacterial load has been proposed to be a useful marker for assessing severity, predicting prognosis, and monitoring clinical response [17]. Patients with a high bacterial load of Mycobacterium tuberculosis complex (MTBC) showed a lower rate of conversion to negative by the 8th week of treatment than those with a lower bacterial burden, and the virulence of MTBC isolates in the macrophage lysis model was positively associated with the MTBC DNA load in sputum of patients [18,19]. In meningococcal disease, a higher DNA load of *Neisseria meningitidis* is associated with an increased risk of death, prolonged hospitalization, and infection with the more virulent serogroup C [20].

Although studies have examined the relationship of DNA load and disease severity for surviving and non-surviving patients with scrub typhus, the association between DNA load and severity in surviving patients has not been investigated [15,16]. In this study, the *O*. *tsutsugamushi* genomic load in blood samples of patients with scrub typhus taken at the time of hospital admission was evaluated, and its association with disease severity, *O*. *tsutsugamushi* genotypes, and other clinical features was analyzed.

## Methods

### Ethics statement

This study was approved by the Ethics Committee of Hainan Medical University. Written informed consent to participate in this study was obtained from adult patients. For patients younger than 18 years, the written informed consent was obtained from their parent or legal guardian.

### Patient recruitment and case definition

Patients presenting at the infectious disease departments with acute undifferentiated fever (FUO) were recruited from 4 hospitals in Hainan Province from July 2018 to November 2021. The hospitals included the First Affiliated Hospital of Hainan Medical University, the Second Affiliated Hospital of Hainan Medical University, the People’s Hospital of Haikou City from Haikou, which is the provincial capital, and the People’s Hospital of Qiongzhong Li Miao Autonomous County, located at the center of Hainan Province. Patients who fulfilled the following criteria were enrolled in this study: 1) axillary temperature ≥ 37.5°C accompanied by at least 1 of these symptoms (eschar, skin rash, lymphadenopathy, hepatomegaly, and/or splenomegaly); and 2) history of field exposure within 3 weeks before the onset of symptoms.

Whole blood and serum samples were collected immediately following admission. The samples were transported to the laboratory within 48 h using cold chain transportation and stored at -80°C before testing. The clinical characteristics and demographic features of the enrolled patients were also collected.

Scrub typhus severity was grouped by the number of dysfunctional organs, with mild, moderate, and severe corresponding to no organ dysfunction, one-organ dysfunction, and two-organ or more dysfunction, respectively, as previously described [21,22]. Briefly, organ dysfunction was defined as follows: 1) Pulmonary dysfunction: bilateral pulmonary shadows on chest X-rays with moderate or severe hypoxia (PaO2/FiO2 < 250 mmHg or PaO2 < 60 mmHg or SpO2 < 90%) or mechanical ventilation requirement, 2) Cardiovascular dysfunction: cardiovascular systolic blood pressure < 80 mmHg despite fluid resuscitation or severe anemia (hemoglobin < 60 g/L) or a diagnosis of myocarditis/myocardial ischemia/arrhythmia/heart failure, 3) Central nervous system dysfunction: a clinical diagnosis of epilepsy/cerebral hemorrhage/cerebral infarction/meningitis or coma, 4) Hepatic dysfunction: bilirubin (total) ≥ 42.7μmol/L, 5) Renal dysfunction: creatinine ≥177μmol/L, and 6) Digestive dysfunction: a diagnosis of gastrointestinal hemorrhage

### Nucleotide sequence and phylogenetic analyses

Genomic DNA was extracted from whole blood samples using a QIAamp DNA Mini Kit (QIAGEN, Hilden, Germany) according to the manufacturer’s instructions. Nested PCR was performed using primers targeting a 483bp fragment of the 56-kDa TSA gene as previously reported [23]]. The amplified product of nested PCR was purified using a QIAquick gel extraction kit (Qiagen) and sent to Sangon Biotech (Sangon, Shanghai) for Sanger sequencing with a 3730×1 DNA analyzer (Applied Biosystems, Foster, CA, USA) in both forward and reverse directions. The 2 obtained sequences of the 56 kDa TSA gene from the same samples were aligned using DNASTAR SeqMan II to obtain a confirmed sequence for further analyses. The resulting sequences (GenBank accession No. ON862382—ON862498) were aligned with the basic local alignment search tool (BLAST) using the National Center for Biotechnology Information (NCBI) database to determine the closest sequences. The newly obtained nucleotide sequences were aligned with their closest related sequences and representative sequences from the worldwide NCBI database using CLUSTAL X software. Phylogenetic analysis was performed using the neighbor-joining method with a bootstrap of 1000.

### Quantitative real-time PCR and *O*. *tsutsugamushi DNA load*

The 56 kDa TSA protein, a major antigenic determinant, is widely used in the diagnosis and quantification of *O*. *tsutsugamushi* infection by real-time PCR [24,25]. In this study, a quantitative real-time PCR assay was developed based on the *56 kDa TSA* gene of *O*. *tsutsugamushi* (GenBank accession numbers: AY357216, AF050669, AY222631, AY222635, and M33004). The dual-labeled probe (TaqMan) and primers were designed using primer5, and the sequences were as follows: 5′ TGATAAGGATATTAAAGGGCATA 3′ (Ot56KDa-F), 5′ ATACACCCTCAGCAGCATTAAT 3′ (Ot56KDa-R), and 5′ [6-carboxyfluorescein]-ATGGTTGCATCAGGAGCACTTGG-[BHQ1] 3′ (Ot56KDa-Probe). The real-time PCR was performed in a 20-μL reaction mixture containing 5 μL template DNA, 1 μL (10 μmol/L) of each primer, 1 μL (10 μmol/L) probe, 10 μL 2x Fast Probe Mixture (Fast Probe Mixture, China), 0.4 μL 50×Low ROX, and 2.6 μL of distilled water. The reaction conditions consisted of an initial activation at 95°C for 5 min, followed by 40 cycles of 5 s at 95°C and 10 s at 60°C.

### Statistical analysis

Statistical analyses were performed using SPSS software version 19.0, and figures were generated using GraphPad Prism 6.0. Nonparametric tests (Kruskal–Wallis test) were used to compare the differences between bacterial DNA copies and the indicated groups. The duration of illness and the highest body temperature recorded during fever among the groups with different disease severity were compared by one-way analysis of variance (ANOVA) tests. Statistical significance was set as *P* < 0.05. Correlation analyses were performed using Spearman correlation.

## Results

### Variation of *O*. *tsutsugamushi DNA* load in patients with scrub typhus

**Fig 1:**
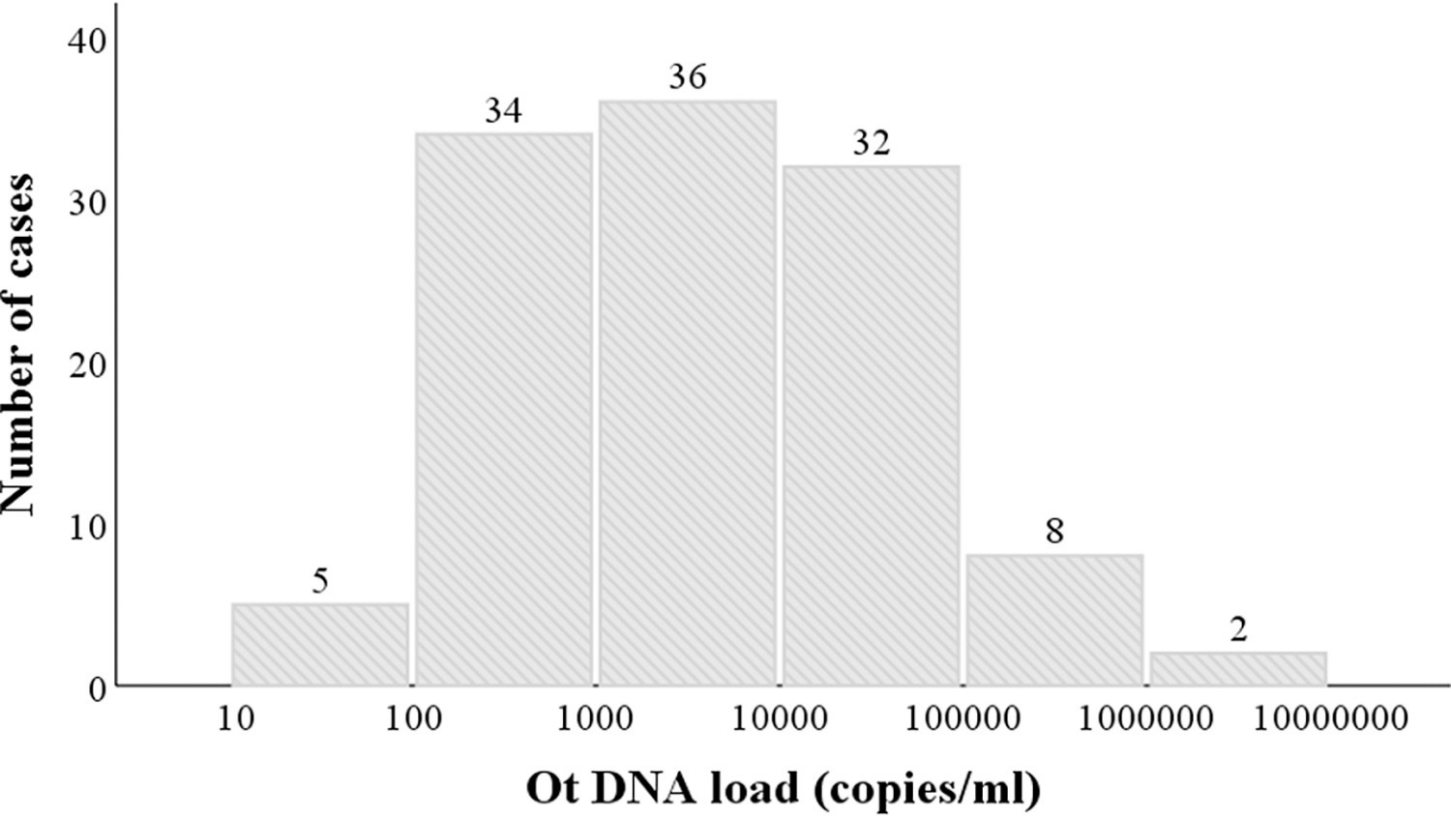
Histogram of the calculated *O*. *tsutsugamushi* DNA loads (numbers of DNA copies/ml blood) in 117 patients with scrub typhus who have not received appropriate antibiotic treatments.

From 2018 to 2021, 680 blood samples were collected from hospitalized patients with FUO for this prospective observation study. One hundred and forty-two patients were positive for nested PCR, accounting for 21% (142/680) of the FUO patients. Real-time PCR was performed on the 142 nested PCR positive patients, and 135 cases had detectable bacterial loads with detection rate of 95% (135/142), accounting for 20% (135/680) of the FUO patients. The median (interquartile range [IQR], range) *O*. *tsutsugamushi* DNA load was 2.92×10^3^ (442–21824, 66–3.3×10^6^) copies/ml. As *O*. *tsutsugamushi* is sensitive to treatment with the appropriate antibiotics, to exclude the interference of treatment, 117 samples from patients who had not received prior antibiotic treatments (screened using the medical history information in their medical records), including doxycycline, tetracycline, chloramphenicol, rifampicin, or azithromycin, were selected for further analyses. The most common symptoms of the 117 patients included fever (117/117, 100%) followed by eschar or skin ulcer (44/117, 38%). Other nonspecific symptoms included chill, headache, fatigue, cough, lymphadenopathy, and generalized myalgia (Table 1). The median (IQR) *O*. *tsutsugamushi* DNA load of the 117 patients was 3.11×10^3^ (470–21616) copies/mL, ranging between 44–3.3×10^6^ copies/mL (Fig 1). Most (87%) patients had an *O*. *tsutsugamushi* DNA load ranging between 1×10^2^ and 1×10^5^ copies/mL, of which 34 cases had a DNA load ranging from 10^2^ to 10^3^ copies/mL, 36 cases ranged between 10^3^ and 10^4^ copies/mL, and 32 cases ranged between 10^4^ and 10^5^ copies/mL. Only 13% of the patients had a DNA load of ≤ 10^2^ copies/mL or ≥ 10^5^ copies/mL.

**Table 1:** Clinical presentation of patients with scrub typhus.

Symptoms	Number of case	Percent (%)
Fever	117	100
Chill	53	45
Eschar	44	37
Headache	43	36
Fatigue	41	36
Cough	37	31
Dizziness	22	20
Generalized myalgia	21	18
Expectoration	17	15
Shiver	16	14
Nausea	10	9
Lymphadenopathy	10	9
Abdominal pain	8	7
Anhelation	6	5
Chest distress or Pectoralgia	5	4
Emesis	5	4
Skin rash	5	4
Tonsillitis	3	3

### Relationship between *O*. *tsutsugamushi* DNA load and genotype

**Fig 2:**
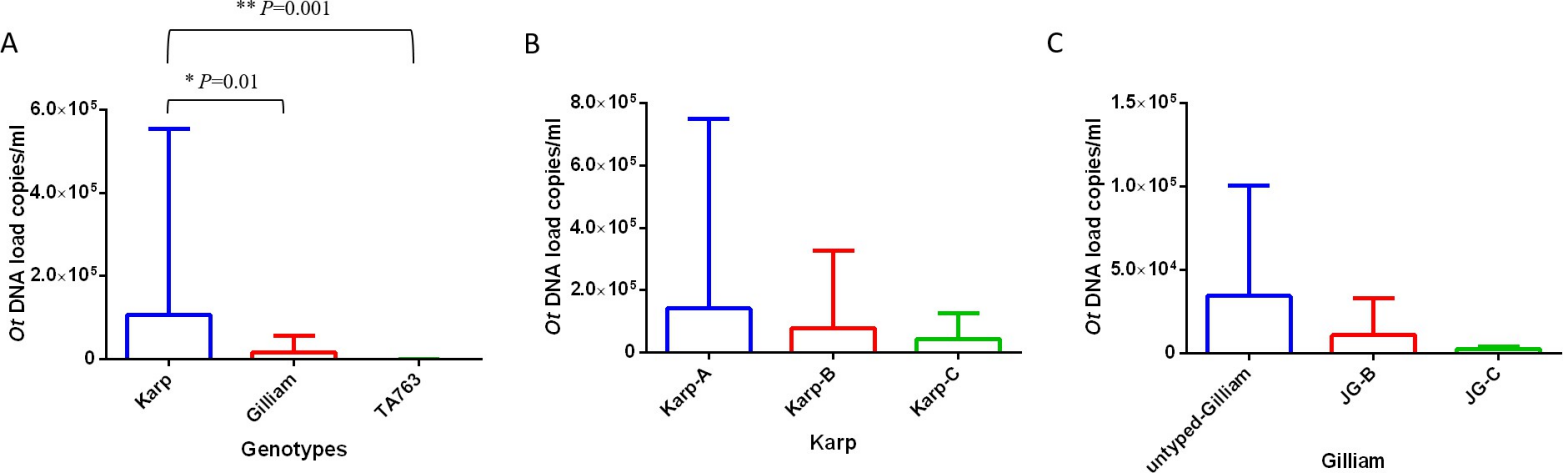
Relationship between *O*. *tsutsugamushi* DNA load and genotype. *P* values among groups were calculated by Kruskal-Wallis test; * represented *P *< 0.05; ** represented *P *< 0.01.

Phylogenic analysis was performed based on truncated *56 kDa TSA* protein gene sequences amplified by nested PCR (S1 Fig). The results indicated that among the 117 cases, 64 were closely related to the Karp group, with sub-genotypes A, B, and C contributing to 29, 15, and 20 cases, respectively. Forty-four cases were closely related to the Gilliam group, comprising 13 and 27 cases with contributions from the JG-B and JG-G sub-genotypes, respectively, and 4 cases of untyped Gilliam. Seven cases were closely related to the TA763 group, all of which were TA763 A sub-genotypes. The DNA loads of the 3 groups were compared, and the results indicated that the DNA load of patients in the Karp group was significantly higher than that in the Gilliam (mean [SD] values: 106926.61 [446370.52] copies/mL versus 16994.09 [40620.40] copies/mL, *P* = 0.01) and TA763 groups (mean [SD] values: 106926.61 [446370.52] copies/mL versus 470.14 [762.32] copies/mL, *P* = 0.001) (Fig 2A). No significant difference was observed between the Gilliam and TA763 (*P* = 0.07) groups (Fig 2A). The DNA loads of the samples between sub-genotypes were further analyzed, and there was no significant difference between sub-genotypes within the Karp (*P* = 0.21) or Gilliam groups (*P* = 0.059) (Fig 2B and 2C).

### The Relationship between *O*. *tsutsugamushi* DNA load and general characteristics of patients with scrub typhus

**Fig 3:**
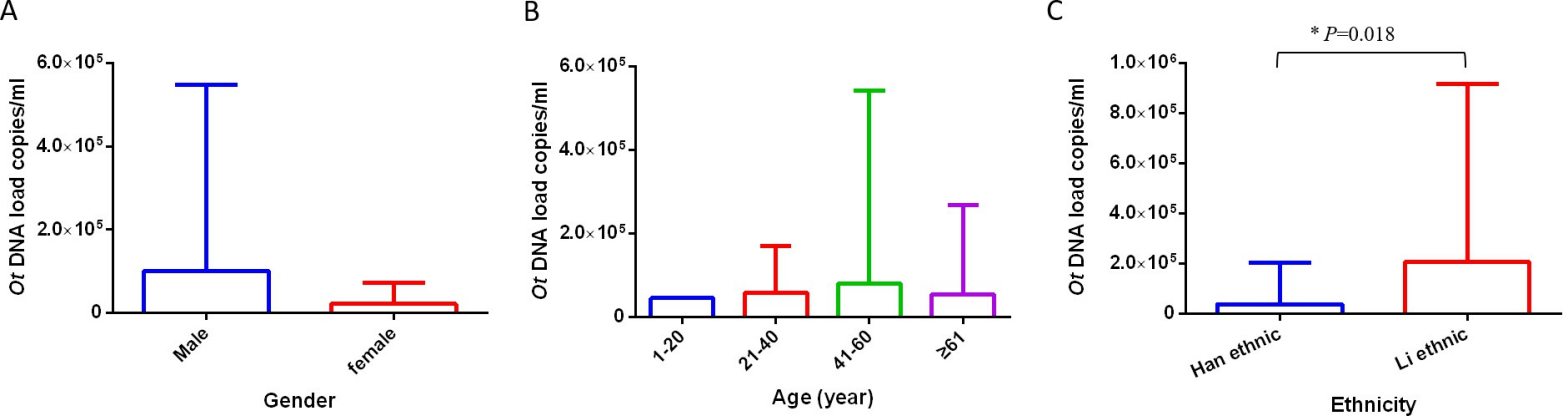
**Relationship between *O*. *tsutsugamushi* DNA load and gender (A), age (B) and ethnicity (C) of patients with scrub typhus.** *P* values among groups were calculated by Kruskal-Wallis test; * represented *P *< 0.05.

Among the 117 patients, 55% (64/117) were men and 45% (53/117) were women. The mean age of the patients was 55.88 *±* 14.33 years, with 1 patient aged < 20 years (10 years old). Among the patients, 43% were 41–60 years old, which was the largest age group, followed by 41% of patients aged > 61 years. Eighty-three of the cases belonged to the Han Chinese ethnic group, 21 belonged to the Li ethnic group. The patients were grouped according to sex, age, and ethnicity, and the DNA load was compared among the groups (Fig 3A, 3B and 3C). The DNA load of patients in the Li group was significantly higher than that of the Han ethnic groups (median DNA load of 8145 copies/mL versus 2177 copies/mL, *P* = 0.018) (Fig 3C). There was no significant difference in the *O*. *tsutsugamushi* DNA load among patients of different sexes (*P* = 0.48) and ages (*P* = 0.60) (Fig 3A and 3B).

### The association between *O*. *tsutsugamushi* DNA load and clinical characteristics

**Fig 4:**
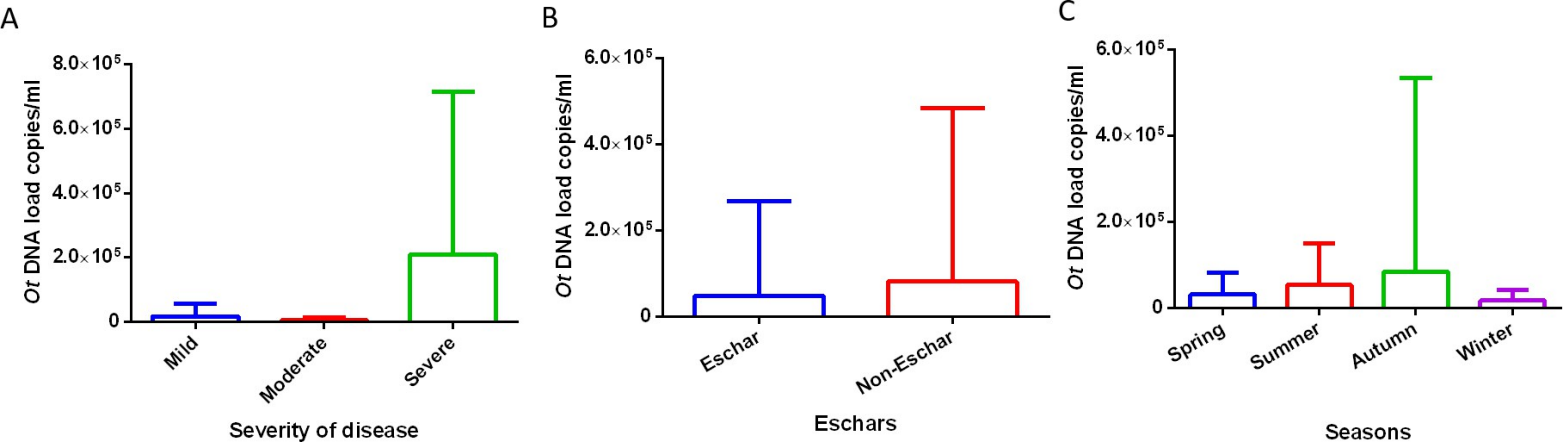
**Relationship between *O*. *tsutsugamushi* DNA load with severity of disease (A), eschars (B) and disease onset (C).** *P* values among groups were calculated by Kruskal-Wallis test; * represented *P *< 0.05, ** represented *P *< 0.01.

Detailed medical documentation was available for 77 patients with positive RT-PCR, based on which these cases were categorized as mild (N  =  54), moderate (N  =  15), or severe (N  =  8). Although the median value of *O*. *tsutsugamushi* DNA load in the severe group is considerably higher than that in the moderate (mean [SD] values: 210,094.25 [505,200.32] copies/mL versus 5,938.22 [8,908.11], *P* = 0.11) and mild groups (mean [SD] values: 210,094.25 [505,200.32] copies/mL versus 17,910.41 [38,752.41] copies/mL), these differences were not statistically significant (*P* = 0.11 and *P* = 0.21, respectively) (Fig 4A). The duration of illness and the highest body temperature recorded during fever was also compared and no significant difference was found among the three groups with different disease severity. In addition, we compared the differences in DNA load between patients with (N  =  44) and without eschars (N  =  67), and the results showed no significant differences between the 2 groups (Fig 4B). Patients were divided into 4 groups based on the season of disease onset: spring (March–May), summer (June–August), autumn (September–November), and winter (December–February). A comparison of the *O*. *tsutsugamushi* DNA load in patients indicated that there was no significant difference among the 4 groups (*P* = 0.09) (Fig 4C). The correlation between DNA load and duration of illness (N = 117) and the highest body temperature recorded during fever (N = 77) was also evaluated using correlation analyses. The results indicated that only the highest body temperature (ρ  =  0.272, *P * =  0.022) was positively correlated with *O*. *tsutsugamushi* DNA load (Table 2).

Changes in blood profile have been proposed as being related to the severity of scrub typhus. Therefore, we analyzed the correlation of white blood cell count (N  =  85), lymphocyte ratio (N  =  44), neutrophil ratio (N  =  64), platelet count (N  =  47), alanine aminotransferase (N  =  55), aspartate aminotransferase (N  =  53), total bilirubin (TBil) (N  =  28), and creatinine (N  =  24) with DNA load. The results showed that TBil was positively correlated with DNA load (ρ  =  0.454, *P * =  0.015), whereas other tests showed no significant correlation (Table 2).

**Table 2:** Correlation between *O*. *tsutsugamushi* DNA load and characteristics of clinical examination of patients with scrub typhus.

Variables	^α^Association with *Ot* DNA load	
	ρ	*P-*value
Body temperature	0.272	0.022*
Duration of disease	-0.112	0.227^NS^
Leukocyte count, 10^9^/mL (WBC)	0.014	0.902^NS^
Lymphocyte, %(LY%)	-0.167	0.279^NS^
Neutrophil, %(NEUT%)	0.155	0.218^NS^
Platelet count, 10^9^/mL	-0.007	0.961^NS^
Alanine aminotransferase concn, U/L(ALT)	0.035	0.796^NS^
Aspartate aminotransferase concn, U/L(AST)	0.160	0.247^NS^
Total bilirubin level, μmol/L (TBil)	0.454	0.015*
Serum creatinine concn, μmol/L	0.337	0.107^NS^

α were calculated by Spearman’s rho.

ρ correlation coefficient

*Indicates statistical significance

NS is not significant.

## Discussion

High bacterial load is an indicator of disease severity in many infections; however, the association between *O*. *tsutsugamushi* DNA load and disease severity has not been widely investigated. In this study, we analyzed the association of DNA load with disease severity, *O*. *tsutsugamushi* genotype and clinical factors in 117 patients who had not received appropriate antibiotic treatment. Our results indicated that 95.07% of nested PCR positive patients had detectable bacterial loads in real-time PCR assays. Several real-time PCR assays have been developed for the detection and quantitative analysis of *O*. *tsutsugamushi* infection, in which 16S rRNA genes and the membrane protein 56-kDa and 47-kDa genes are the most widely used targets [15,16,26]. In this study, we developed a real-time PCR assay for the quantification of *O*. *tsutsugamushi* targeting the 56-kDa TSA gene of *O*. *tsutsugamushi*, and the assay showed good concordance with the nested PCR assay.

The median *O*. *tsutsugamushi* DNA load in this study was 3.11×10^3^ copies/mL, ranging between 44 and 3.2×10^6^ copies/mL. However, this load varies among studies; Singhsilarak *et al*. reported a median number of 4.2×10^6^ copies/mL (range, 1×10^6^–2.88 ×10^7^ copies/mL) using real-time PCR targeting the 47 kDa gene in 7 patients with scrub typhus and positive RT-PCR, whereas Kim *et al*. reported a median number of 7.8×10^4^ copies/mL (range 3,960) in surviving patients, and a median number of 8.38×10^7^ copies/mL (range 244,600) in 3 non-surviving patients using the same primers and probes [15,16]. Sonthayanon *et al* reported a median number of 284 copies/mL of *O*. *tsutsugamushi* DNA (range, 124–943 copies/mL) in 81 patients with positive quantitative RT-PCR results targeting 16 S rRNA [15]. In addition to population, chigger species, and *O*. *tsutsugamushi* strains, variation in bacterial loads was proposed to be partially affected by the target genes selected for the RT-PCR assay and inclusion criteria of these studies [15].

The mortality rate of scrub typhus varies greatly among regions studied, and the cause of this variation remains largely unknown; however, the distribution of *O*. *tsutsugamushi* genotypes was proposed to play an important role in this variation [16]. Karp and closely related strains are found in most endemic countries, including Japan, Korea, China, Thailand and Southeast Asia [12,13,27]. The dominance of sub-genotypes varies among countries: Boryong is predominant in South Korea, Karp_C in Japan, and Karp_A in Taiwan, Thailand and Cambodia [13,27]. Several sub-genotypes of the Gilliam group are also prevalent in endemic countries: the Kawasaki genotype is prevalent in South Korea, and the JG-C genotype is primarily found in Taiwan, Thailand and Cambodia [27]. Isolates belonging to the TA763 and Kato groups were mainly reported in Taiwan [27]. The characteristics of the genetic components of each genotype can influence the ability of *O*. *tsutsugamushi* to invade the host, grow in large numbers, and disseminate. The severity and clinical presentation of scrub typhus were found to be strain-dependent in both humans and laboratory animals [7,28]. Differences in clinical features between prototypes of *O*. *tsutsugamushi* have also been reported. Patients infected by the Boryoung cluster had significantly more generalized weakness, eschars, skin rashes, conjunctival injection, high albumin levels, and greater ESR and fibrinogen levels than those infected with the Karp cluster; however, the treatment response to current antibiotics was significantly slower in the Karp cluster than in the Boryoung cluster [14]. Additionally, the Karp genotype was proposed as a severe genotype. Consistently, our results indicate that Karp is correlated with the severe clinical characteristics of patients. The DNA load of the Karp genotype was significantly higher than that of the Gilliam and TA763 genotypes, indicating a more efficient propagation of *O*. *tsutsugamushi* and a slower immune clearance in infections with the Karp genotype. Similar results were reported in C57BL/6NJcl mice showing a higher bacterial load of the Karp genotype compared to the less virulent strain UT176 [29].

Genetic differences in host populations may influence susceptibility and responses to *O*. *tsutsugamushi* infection and are proposed to be another factor that influences DNA load. Single-nucleotide polymorphisms (SNPs) in TLR2, TLR4, and HSP70 genes, which lead to variations in immune response and alter susceptibility to various infectious diseases, were analyzed in the Tamil-speaking Dravidian ethnic group in India, and the prevalence of the TLR4D299G SNP was found to be significantly higher in patients with scrub typhus than in controls [30]. A genome-wide association study (GWAS) in Korean patients with scrub typhus identified 8 potent scrub typhus-related SNPs located on *PRMT6*, *PLGLB2*, *DTWD2*, *BATF*, *JDP2*, *ONECUT1*, *WDR72*, *KLK*, *MAP3K7*, and *TGFBR2* genes [31]. A study in mice using the Gilliam strain found that *O*. *tsutsugamushi* infections showed significantly different degrees of virulence due to genetic differences between mouse strains [32]. Additionally, a comparison of lethal and nonlethal mouse models of *O*. *tsutsugamushi* infection revealed differences in CD4+ and CD8+ T-cell population-associated cytokines between the 2 groups [33]. Notably, in the present study, patients in the Li ethnic group showed significantly higher DNA load than the Han groups. To verify whether the difference was caused by the infecting *O*. *tsutsugamushi* genotypes, the composition of the genotypes in Han and Li ethnic groups was analyzed. The main genotypes of Han ethnic groups (N = 51) were Karp (N = 30, 59%), Gilliam (N = 18, 35%), and TA763 (N = 3, 6%), while those of Li ethnic groups (N = 21) were Karp (N = 13, 62%) and Gilliam (N = 8, 38%). Statistical analysis showed that the genotypes of the two ethnic groups were not significantly different. Additionally, 95% (20/21) of the Li ethnic patients were recruited in the Qiongzhong Li Miao Autonomous County. The county is located at the center of Hainan Island and most of the area is mountainous with forests. Most patients of the area are forest workers or rubber workers. In contrast, patients recruited in Haikou were mostly from the coastal cities and the vegetation varied from Qiongzhong. Patients from Haikou also had various occupations including farmers and factory workers. These differences in ecological and behavioral factors may also contribute to the difference in *O*. *tsutsugamushi* DNA load between patients of Li and Han ethnic groups. It would be interesting to assess the existence of SNPs in these patients that might explain the variations in the immune response and susceptibility to *O*. *tsutsugamushi* infection.

Sonthayanon *et al*. reported that hepatic enzymes are positively correlated with *O*. *tsutsugamushi* DNA load in patients recruited in northeast Thailand [15]. In contrast, we found no significant difference in *O*. *tsutsugamushi* DNA load between the severe, moderate, and mild groups. This is consistent with the finding of no correlation between hematological indicators and DNA load, with the exception of TBil being positively related to DNA load, which is indicative of hepatic impairment. Eschar is a typical clinical sign of scrub typhus, and the prevalence of eschar in patients has been reported to be highly variable (7%–80%) and largely dependent on the *O*. *tsutsugamushi* genotype [14,34]. The presence of eschar was reported to be correlated with DNA load in the study by Sonthayanon *et al*.; however, although eschar was observed in 40.5% (45/111) of patients with physical examination data available in this study, no significant difference was found between patients with and without eschar [15]. The differences in the correlation analyses of DNA load with clinical characteristics between this study and that by Sonthayanon *et al*. may be due to multiple factors. The population in the study reported by Sonthayanon *et al*. in northeast Thailand was predominantly Thai, whereas our study population was predominantly Han and Li ethnic groups. Additionally, the differences could be related to the chigger. The main vectors of scrub typhus in Thailand were reported to be *Leptotrombidium imphalum*, *L*. *deliense*, and *L*. *chiangraiensis*. In contrast, although the vectors in Hainan Island were not studied widely, the mainly reported vector was *L*. *deliense* [35,36]. Bitten by different species of chiggers could result in the difference in clinical characteristics in both human and mice, which may also related to *O*. *tsutsugamushi* DNA load [37,38].

The major limitation of the study was that a small number of cases with scrub typhus were include. In addition, only 77 patients had detailed medical documentation. Another limitation of the study was that only some of the cases had a detailed blood profile comprising all the indicators shown in Table 2, as the clinical test items varied among patients with different symptoms. This may have led to bias in the results obtained from correlation analyses. A larger sample cohort with more clinical data is recommended in future studies to explore the relationship between *O*. *tsutsugamushi* DNA load and clinical characteristics in patients with scrub typhus.

## Conclusion

In conclusion, our study demonstrated that genomic DNA load of *O*. *tsutsugamushi* was associated with its genotypes, ethnic group of the patients, the highest body temperature recorded during fever onset and the total bilirubin (TBil) in the blood. However, no significant difference in *O*. *tsutsugamushi* DNA load was found when comparing mild, moderate, and severe patient groups nor when comparing the groups of patients with or without eschar. This research contributes to the understanding of the pathogenic mechanism of *O*. *tsutsugamushi*.

## Supporting information

S1 Fig Phylogenetic analysis of truncated 56 kDa TSA protein gene sequences of *Orientia tsutsugamushi*. Phylogenetic analysis was performed using the software MEGAX. Phylogenetic tree was constructed by the neighbor-joining method with bootstrap of 1000 repeat.
(DOCX)
