# Supplementary material for: Genomic bacterial load associated with bacterial genotypes and clinical characteristics in patients with scrub typhus in Hainan Island, Southern China
Source: PLoS Negl Trop Dis. 2023 Apr 21;17(4):e0011243. doi: 10.1371/journal.pntd.0011243 (PMC10155967; doi:10.1371/journal.pntd.0011243)

Supplementary figure 1. Phylogenetic analysis of truncated 56 kDa TSA protein gene sequences of *Orientia tsutsugamushi*. Phylogenetic analysis were performed using the software MEGAX. Phylogenetic tree was constructed by the neighbor-joining method with bootstrap of 1000 repeat.


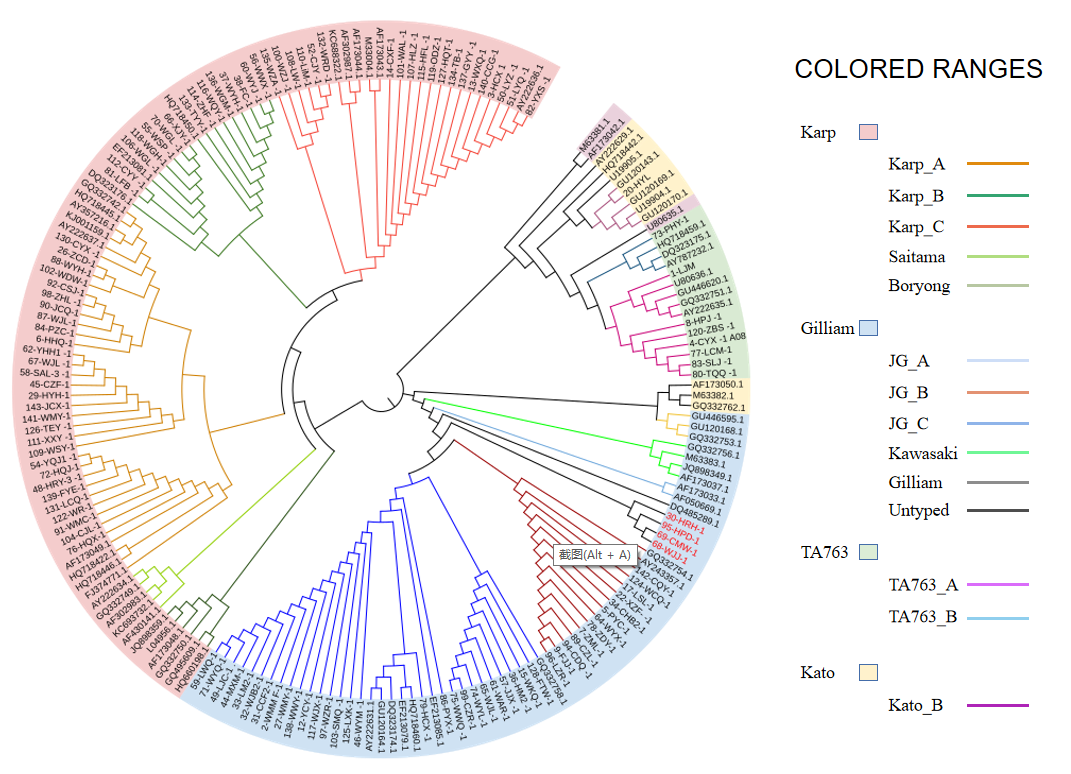

Supplement: S1 Fig — Phylogenetic analysis was performed using the software MEGAX. Phylogenetic tree was constructed by the neighbor-joining method with bootstrap of 1000 repeat. (DOCX) [file pntd.0011243.s001.docx]
